# Research on High Layer Thickness Fabricated of 316L by Selective Laser Melting

**DOI:** 10.3390/ma10091055

**Published:** 2017-09-08

**Authors:** Shuo Wang, Yude Liu, Wentian Shi, Bin Qi, Jin Yang, Feifei Zhang, Dong Han, Yingyi Ma

**Affiliations:** School of Material and Mechanical Engineering, Beijing Technology and Business University, Beijing 100048, China; w.shuo2009@gmail.com (S.W.); shiwt@th.btbu.edu.cn (W.S.); qibinest@foxmail.com (B.Q.); y_jin93@outlook.com (J.Y.); z_feifei90@outlook.com (F.Z.); tssilverbullet@hotmail.com (D.H.); yingyima@foxmail.com (Y.M.)

**Keywords:** selective melting laser, high layer thickness fabricated, fine powder, building rate, microstructure, tensile properties

## Abstract

Selective laser melting (SLM) is a potential additive manufacturing (AM) technology. However, the application of SLM was confined due to low efficiency. To improve efficiency, SLM fabrication with a high layer thickness and fine powder was systematically researched, and the void areas and hollow powders can be reduced by using fine powder. Single-track experiments were used to narrow down process parameter windows. Multi-layer fabrication relative density can be reached 99.99% at the exposure time-point distance-hatch space of 120 μs-40 μm-240 μm. Also, the building rate can be up to 12 mm^3^/s, which is about 3–10 times higher than the previous studies. Three typical defects were found by studying deeply, including the un-melted defect between the molten pools, the micro-pore defect within the molten pool, and the irregular distribution of the splashing phenomenon. Moreover, the microstructure is mostly equiaxed crystals and a small amount of columnar crystals. The averages of ultimate tensile strength, yield strength, and elongation are 625 MPa, 525 MPa, and 39.9%, respectively. As exposure time increased from 80 μs to 200 μs, the grain size is gradually grown up from 0.98 μm to 2.23 μm, the grain aspect ratio is close to 1, and the tensile properties are shown as a downward trend. The tensile properties of high layer thickness fabricated are not significantly different than those with a coarse-powder layer thickness of low in previous research.

## 1. Introduction

Selective laser melting (SLM) is a type of promising metal additive manufacturing (AM) technology, in which functional complex parts can be formed into arbitrary shapes by melting layers of powder particle selectively and successively without traditional processing [1,2,3]. SLM has a new potential development of the most innovate laser manufacturing technology, which has been widely used in aerospace, medicine, and automotive fields because of generated metal parts with fine surface roughness, high relative density, high mechanical properties, and even arbitrary complex structures [4,5,6,7,8,9,10,11,12].

Generally, during the SLM fabrication process, the formation of low layer thickness can result in high surface precision [13,14,15,16,17,18,19]. Kamath et al. investigated the selective laser melting 316L low layer thickness (30 μm) formation process, in which high relative density can be reached 99.81% [20]. Cherry et al. report that the high relative density of 99.62% and the surface roughness can be reached R_a_9 with a powder layer thickness of 50 μm [21]. Also, Sun et al. reported SLM fabrication of 316L with the layer thickness of 50 μm, which was aimed at obtaining a high relative density of 99.9% [22]. These results confirmed that the low layer thickness fabricated can result in high relative density and high precision through previous studies. However, the application of SLM technology is still confined due to its fabrication process with low layer thickness (about 30 μm to 50 μm generally), which results in poor efficiency and a reduction in the ability to produce large scale metal parts. Increasing layer thickness is considered to be a key technology for improving the building rate of fabrication metal parts. Later, Ma et al. increased the layer thickness from 60 μm to 150 μm for the production of 1Cr18Ni9Ti parts; the block sample relative densities can be reached a range from 99.3% to 99.8%, and the building rate can be increased 10–20 times [23]. Shi et al. investigated the SLM of the Ti-6Al-4V formation process with high layer thickness, which can obtain the approximation full-relative-density block, and the building rate can be improved to 7.2 mm^3^/s [24]. The previous studies have been demonstrated that high layer thickness fabricated is an effective approach to improve the building rate. Up to now, the high layer thickness of TC4 fabricated has been obtained by the approximation full relative density sample, and the high layer thickness of 316L, as one of the most common metal materials, has not been systematically researched. It is worth noting the difference between the metallurgical bond, the microstructure, and the mechanical properties when comparing the high layer thickness fabricated with the low layer thickness via SLM.

Hence, in this research, SLM, with a thicker powder layer of 150 μm and an average particle (D_50_) of 18 μm fine powder, is researched. Consisting of single-track experiments and multi-layer fabrications, the SLM fabrication process is systematically researched to obtain high relative densities. Additionally, the influences of process parameters on relative density, microstructure, and mechanical properties have been analyzed, and the mechanism of defect forming is revealed.

## 2. Materials and Methods

### 2.1. Materials

**Figure 1:**
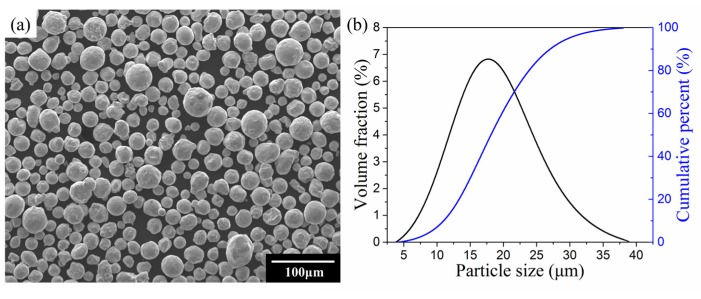
(**a**) Morphology of 316L powder; (**b**) particle size distribution of 316L powder.

The gas-atomized 316L powder with a size range of 5 μm–40 μm supplied by Renishaw plc (London, UK) was employed in this research, and the chemical composition can be depicted as in Table 1. Figure 1a through Figure 1b provides a characteristic of the powder particle based on the observation of the morphology of 316L with normal distribution of D_10_:11.05 μm, D_50_:18.42 μm, D_90_:27.83 μm by scanning electron microscope (SEM) JSM6490 (JEOL, Tokyo, Japan).

### 2.2. Experimental Equipment

**Figure 2:**
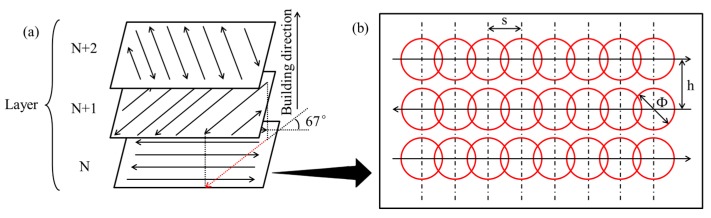
(**a**) Scanning strategy; (**b**) spot-to-spot fabrication process, where “s” is point distance, “h” is hatch space, and “Φ” is laser beam spot size.

SLM experiments were performed on Renishaw AM400 (Renishaw plc, London, UK). A Nd:YAG laser with a maximum power of 400 W and a wavelength of 1075 nm in continuous laser mode was used by the AM400. Figure 2b shows a schematic diagram of the spot-to-spot fabrication process. The process chamber provides a closed environment filled with argon gas, and the Oxygen target can be changed according to different experiments.

### 2.3. Experimental Methods

Single-track experiments, with a length of 10 mm, were carried out as a schematic diagram shown in Figure 2b. To prevent the influences of uneven layer thickness and circulating gas, the same process parameter of single track was performed at a different location twice on the building platform. The single track morphology is affected on exposure time and point distance, so the exposure time was set to 100–160 μs with a step of 40 μs, and the point distance was set to 20–50 μm with a step of 10 μm. Table 2 gives the process parameters of single-track experiments. The morphology of single track was observed by using an optical microscope (OM) DM4000M (Leica, Wetzlar, Germany).

**Figure 3:**
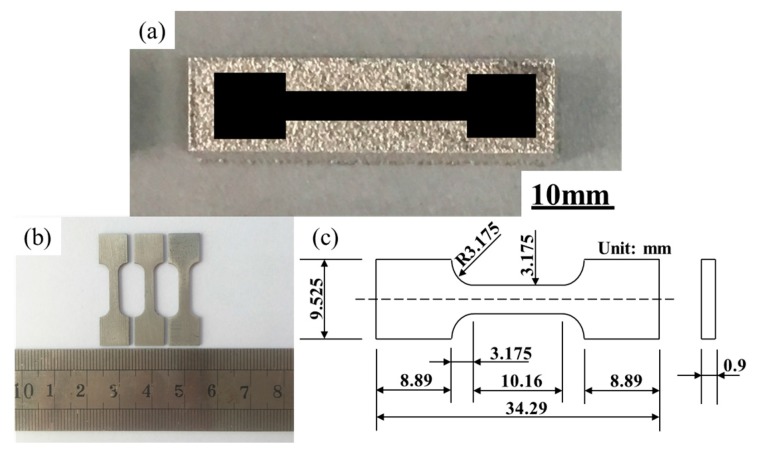
(**a**) Macrograph of the sample; (**b**) tensile specimens; (**c**) geometrical size of tensile specimen.

Four groups of process parameters were selected for multi-layer fabrications with 40 × 10 × 6 mm^3^ (20 layers, as shown in Figure 3a) based on single-track experiments. Also, the hatch space was set to 0.5, 0.6, 0.7, and 0.8 times of the molten width, and the molten pool is based on single-track experiments. Table 3 gives the process parameters of the multi-layer fabrication experiments. Figure 2a sketches these relationships for which the scanning strategy was employed using parallel scan vectors, overlaid at an angle of 67° to the previous deposited layer. This scanning strategy has been proven to be an effective approach to improve surface roughness and reduce defects in SLM [25,26].

All of the samples were sectioned by wire cutting equipment (Cmne, Beijing, China), and then ground and polished following standard metallographic procedures. Metallographic specimens were prepared by standard polishing equipment, which were etched by a solution of 4 mL HF, 4 mL HNO_3_, and 92 mL C_2_H_5_OH. The sample cross section microstructures were observed by using SEM and OM. The sample relative densities were determined by the cross-section method, which is based on the analyzing optical micrographs and Image J software. The sample cross section porosity was measured twice in different location. The sample relative density can be calculated by the percentage of porosity area in the block cross section area. Also, when the two data is averaged, the sample relative density can be obtained. Figure 3a through Figure 3c provides the characteristics of the tensile specimen that is about the specimen of the macrograph, shape, and geometrical size. Also, the tensile specimens were evaluated using Instron 5966 (Instron, Boston, MA, USA) at room temperature. At a displacement rate of 0.01 mm/s, a dynamic strain gauge extensometer was applied to record the data.

## 3. Results and Discussion

### 3.1. Single-Track Experiments

The performance of multi-layer fabrication is mainly based on the morphology of single track. To obtain high relative density samples, the morphology of single track should be a uniform, smooth, and stable molten track.

**Figure 4:**
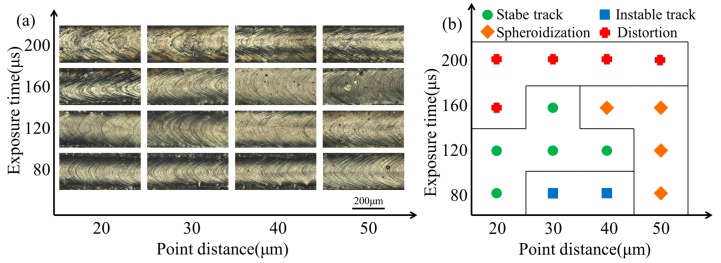
(**a**) Morphologies of single-track experiments; (**b**) process parameter windows of single-track experiments.

Single-track morphologies can be depicted as in Figure 4a. The horizontal coordinate indicates the point distance with the values increasing from 20 μm to 50 μm (a step of 10 μm), and the vertical coordinate indicates the exposure time with the values increasing from 80 μs to 200 μs (a step of 40 μs). The different process parameters match different single-track surface morphologies. For the same point distance, the molten track width was increased with the exposure time increasing. When the exposure time was increased to 200 μs, the molten track of distortion can be observed. The results confirm that the large exposure time leads to the high wettability of the molten pool and spreads to the surrounding irregular. At a point distance of 50 μm, the spheroidization phenomenon was observed with the exposure time increased from 80 μs to 160 μs, for which the line-energy density of single track is relatively lower, and the powder cannot be melted fully. As the exposure time increased from 80 μs to 200 μs, the track width kept expanding from 230.79 μm to 364.41 μm, indicating that molten pool size depended on the exposure time. In addition, the surface tension gradient was produced by the Marangoni effect [27], which may lead to molten pool instability. At the exposure time of 80 μs, the point distances of 30, 40 μm are the unstable molten track. Although the single-track surface is smooth, the metal droplets can be observed on the tracksides. Based on single-track experiments (Figure 4a), the process parameter windows are drawn to present the appropriate process parameters of single tracks, and Figure 4b is indicated by the different symbols. There are four different symbol types of molten track, which can be identified over the entire range of exposure time and point distance. Also, the four forms of single tracks were exhibited with stable track, unstable track, spheroidization, and distortion. The distortion would be caused by a larger exposure time, and the spheroidization would be caused by a larger point distance. The green area is a single track with appropriate process parameters, for the smooth, uniform, and stable molten track.

### 3.2. Multi-Layer Fabrications

#### 3.2.1. Relative Density

Based on the above research, there were five process parameters of the stable molten track. With exposure time as the most important factor of energy input, four different exposure time process parameters were selected with 80 μs, 120 μs, 160 μs, and 200 μs. In addition to improving the poor efficiency, the point distance selected the larger one as much as possible in the range of the stable track area. Hence, the exposure time-point distance of 80 μs-20 μm, 120 μs-40 μm, and 160 μs-30 μm was selected to be applied to fabricate the multi-layer experiments. Also, there was a no stable molten track at the exposure time of 200 μs. Therefore, the process parameter of 200 μs-50 μm was chosen as a comparison group. The samples were fabricated by different exposure times, point distances, and hatch spaces, as shown in Table 3. The influence of the hatch spaces, which is based on the molten width of the single track, was researched.

**Figure 5:**
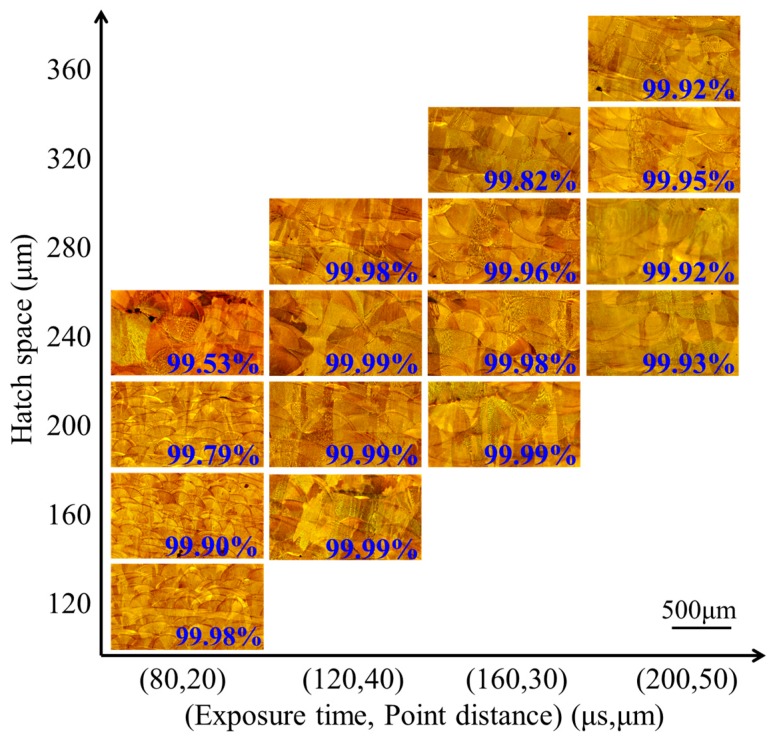
Relative density of multi-layer fabrication.

The multi-layer fabrications of different exposure times, point distances, and hatch spaces on the relative density were depicted as in Figure 5, in which the high-relative-density blocks still reached 99.99% in SLM fabrication. There is a huge influence on relative densities by the exposure times, point distances, and hatch spaces. When the exposure times are from 80 μs to 200 μs, with a hatch space of 240 μm, the relative density ranges are from 99.53% to 99.99%. When the hatch spaces are from 120 μm to 240 μm, with an exposure time of 80 μs, the relative density ranges are from 99.53% to 99.99%. With the exposure times increasing, there is little damage to the hatch spaces on relative densities. At the exposure time of 120 μs, a series of the hatch spaces from 160 μm to 280 μm are nearly influences on relative densities from 99.98% to 99.99%. At the exposure times of 160 μs and 200 μs, the hatch spaces are affected on relative densities when the relative densities are from 99.82% to 99.99% and 99.52% to 99.95%, respectively.

The above results show that high-relative-density samples (99.99%) can be easily obtained within a range of SLM process parameters. However, in previous studies, high layer thickness fabricated was used to produce more pores and low relative densities. The reason mainly is the following two aspects.

**Figure 6:**
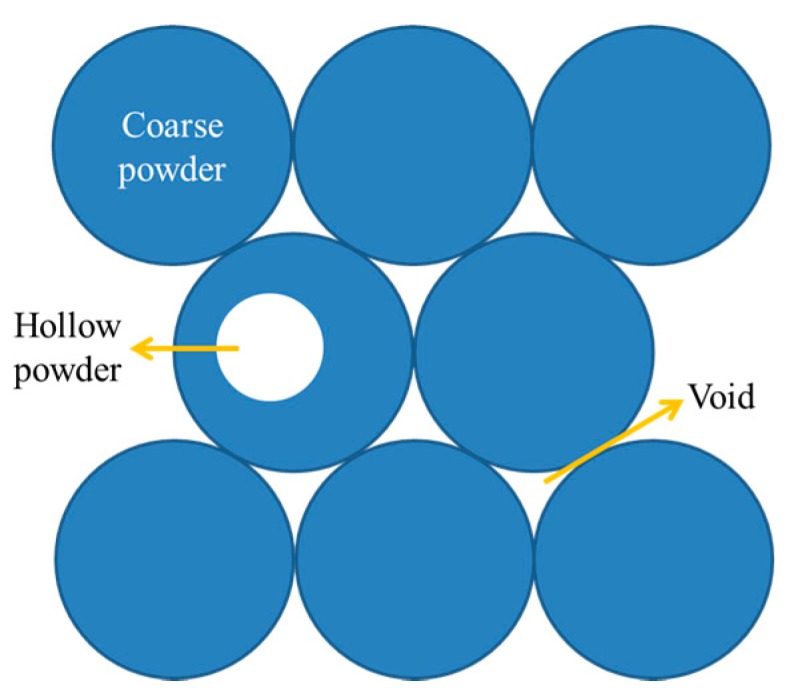
Schematic of coarse powder with hollows and voids.

Firstly, Ma et al. studied a maximum powder layer thickness of 80 μm that can be fabricated with high relative density block; the powder layer thickness increased to 150 μm, and the residual gas at the bottom of molten pool cannot come out in the rapid solidification in a timely manner [23]. There is a significant influence on relative density with high layer thickness fabrication if the coarse powder is employed. The architecture of this layer particle for coarse powder is illustrated in Figure 6 with void area and hollow powder. There is a large void between each particle; meanwhile, the number of hollow powders increased along with the increasing powder particle size [27,28,29,30,31,32,33,34]. Therefore, the layer thickness relative density of coarse powder is relatively low, and more gas components are contained in the powder layer. In Table 4, the particle size is developed and significant recommendations are made. The relative densities can be reached 99% with low layer thickness fabrication on 316L through previous studies. Also, the layer thickness of 30–50 μm and the average powder (D_50_) of 30–60 μm was massively used. Khairallah et al. studied the different powder grades of SLM, and it was confirmed that the fine powder exhibited low particle friction, high mobility, and reduced the pore between the large particles [28]. This research aims to obtain a stable molten pool and an approximation full-relative-density block at a thicker powder layer of 150 μm; thus, the fine powder of average particle (D_50_) of 18 μm was utilized. Fine powder could produce stable molten pools, which ensure that the powder of high layer thickness can be fully melted and the residual gas can come out in time.

Besides, with regard to the equipment factor, the common SLM machines were SLM Solution (Lubeck, Germany), Phenix (Riom, France), EOS (Planegg, Germany), Concept Laser (Lichtenfels, Germany), Renishaw (London, UK), Realizer (Borchen, Germany), and Trumpf (Munich, Germany), which are used in previous studies. Ahmadi et al. used Phenix to study the computational framework by SLM of 316L [16]; Suryawanshi et al. used Concept Laser to study the effects of scanning strategy on mechanical properties [36]; and Mao et al. used EOS to study the manufacturing feasibility of forming Cu-4Sn new material [37]. In this experiment, the use of Renishaw is different from the previous three devices, which split the scanning speed into exposure time and point distance, called ‘’spot-to-spot formation’’, as shown in Figure 2b. Extensive experiments indicate that point distance is divided by exposure time and is approximately equal to the scanning speed. For the same scanning speed, there is a multitude combination of exposure times and point distances. This leads to the fact the same scanning speed of Phenix, Concept Laser, or EOS was behaved to the same molten track morphology, and Renishaw can change the combination of different exposure times and point distances to obtain a more stable molten track, which makes it easier to produce high-relative-density samples during SLM processing.

#### 3.2.2. Building Rate

The forming time of SLM mainly includes auxiliary processing time and laser manufacturing time. The auxiliary processing time is determined by the operation of equipment, including powder delivery time and substrate falling time. Also, the laser manufacturing time is determined by process parameters. Therefore, the laser manufacturing time was shortened mainly by adjusting process parameters. The building rate is defined as:

(1) $$\eta =\frac{\delta \;h\;s}{t},$$

where “η” is building rate (mm^3^/s), “δ” is layer thickness (mm), “*h*” is hatch space (mm), “*s*” is point distance (mm), and “*t*” is exposure time (s). The building rate range of 0.9 mm^3^/s–4.13 mm^3^/s can be realized from previous studies on SLM fabrication of 316L. Table 5 exhibits the summary of previous studies on 316L building rate, for example, Cherry et al. [21] and Kamath et al. The authors of [20] separately used AM250 (Renishaw, London, UK) and M2 (Concept Laser, Lichtenfels, Germany), the building rates of which are 2.48 mm^3^/s and 1.67 mm^3^/s, respectively. The building rate of this research is a range of 4.5 mm^3^/s–14 mm^3^/s. The samples with a relative density of 99.99% were selected, and the building rate can be up to 12 mm^3^/s, which is 3–10 times the previous 316L building rate.

#### 3.2.3. Analysis of Forming Defects

**Figure 7:**
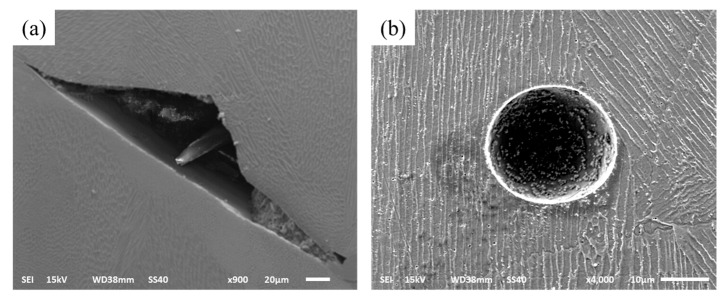
Scanning electron microscope (SEM) micrographs show the different defects (**a**) un-melted defect; (**b**) micropore.

**Figure 8:**
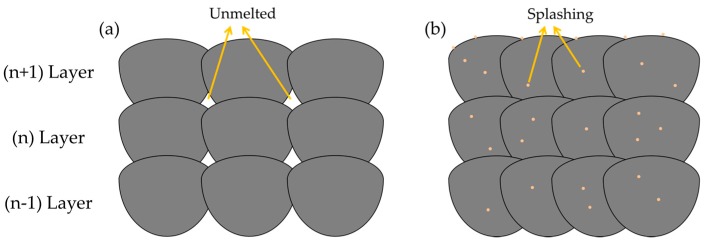
Schematic of selective laser melting (SLM) with (**a**) un-melted defects; (**b**) splashings phenomenon.

**Figure 9:**
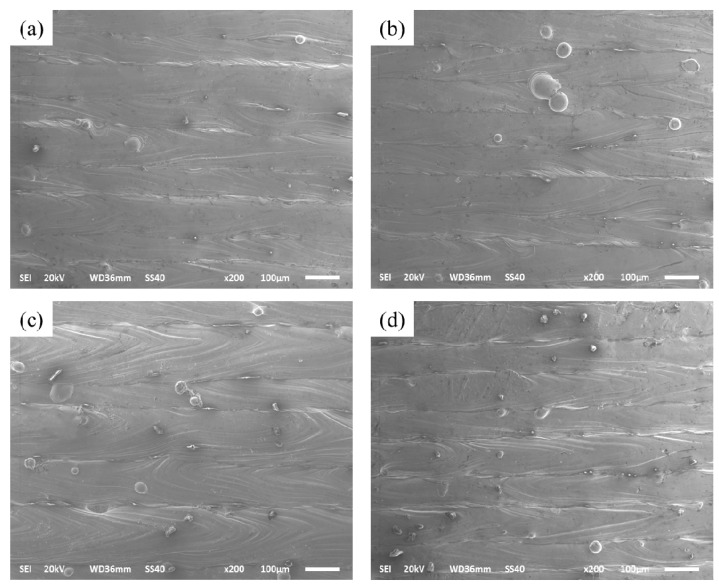
Micrographs showing spheroidization of (**a**) 80 μs; (**b**) 120 μs; (**c**) 160 μs; and (**d**) 200 μs.

In this research, the range of all the sample relative densities is from 99.53% to 99.99% (Figure 5). To reveal the mechanism of forming defects, the specimen cross section was researched. There are three typical defects: the un-melted defect between the molten pools, the micro-pore defect within the molten pool, and the irregular distribution of splashing phenomenon (Figure 7 and Figure 9). When the hatch space is 240 μm at a small exposure time of 80 μs, the overlap rate is less than 20%, the defect shapes are irregular pores, and between the molten pools there is an unmelted area, as shown in Figure 7a. The area of unmelted defect is more than 10,000 μm^2^, having a great influence on the relative density. Consequently, the influence of the hatch space on overlap can result in uneven overlap and surface roughness because of surface deterioration. With the cumulative influence of layer-to-layer fabrication on many rugged depressions, the layers of molten pool cannot be spread fully and produce a larger pore between the molten pools. When the exposure times were increased from 120 to 180 μs, the unmelted defect was gone; at this time, the overlap rates were 40–50%, and the molten track was evenly arranged and overlapped with the adjacent fine overlap. This concept is illustrated in Figure 8a, which is a schematic diagram of un-melted defects. Moreover, there are two reasons: the first is hatch space is determined by molten pool width, the second, molten pool width, is determined by exposure time. Hence, the hatch space is determined by the exposure time. It can be improved for defect-free interfaces at an overlap rate of 40–50%, and the hollow area in the previous solidified layer can be filled. As can be easily understood, the larger unmelted defects are mainly located in the interlayer binding site, resulting in poor metallurgical bonding between layers.

The residual micropore can be depicted within the molten pool as in Figure 7b, in which the spherical pores are very small, with a general area around 100 μm^2^. The location of the micropores is random within the samples, and the formation of spherical pores is not significantly related to process parameters. The experimental results indicated that micropore defects can be found both in the low exposure time of 80 μs and the small hatch space of 120 μm, or the high exposure time of 200 μs and the large hatch space of 360 μm. There are two reasons that some of the micropores are due to the SLM process chamber circulating gas and metal powder fabricated of the hollow powder with residual gas, which was dissolved within the molten pool as the molten pool in liquid phase state, and some of the residual gas at the bottom of the large molten pool failed to come out in time during the rapid solidification. As the exposure times increased from 80 μs to 200 μs, the micropore defects were slowly reduced; on the one hand, it is because a part of the dissolved gases at the bottom of molten pool came out. One the other hand, the other parts of the micropores were scanned secondarily, which also caused the micropores to be released. Therefore, it is considered that some of the micropores are caused by the circulation gas and hollow powder, which was caused not only by the process parameters, but also by the external factors. Optimization of process parameters can partly reduce the micropore phenomenon, but it cannot be completely eliminated.

At an even larger exposure time of 200 μs, there are some adhesions of the small particles on the formation surface with an average area of 100 μm^2^ to 1000 μm^2^. The series of splashings includes spherical splashing, coarse spherical splashing, and irregular splashing [40]. These splashing droplets are cooled and solidified in the process chamber environment to form small metal particles; some of these particles fall onto the surface of the solidified metal, and some of them fall into the molten pool or wrap around the molten pool [41]; Figure 8b gives the outline of a splash phenomenon. Figure 9 indicates the splash phenomenon for different exposure times from 80 μs to 200 μs. It can be observed that the smallest metal particles existed when the exposure time was 80 μs. The laser beam irradiation area temperature is presented as a Gaussian distribution. As the exposure time increased, the center temperature significantly increased, and a significant temperature gradient was produced within the molten pool has. Also, the splash of small particles continually influences the formation of the next deposited layer, reducing the ability to combine with the surrounding metallurgy.

#### 3.2.4. Microstructure

**Figure 10:**
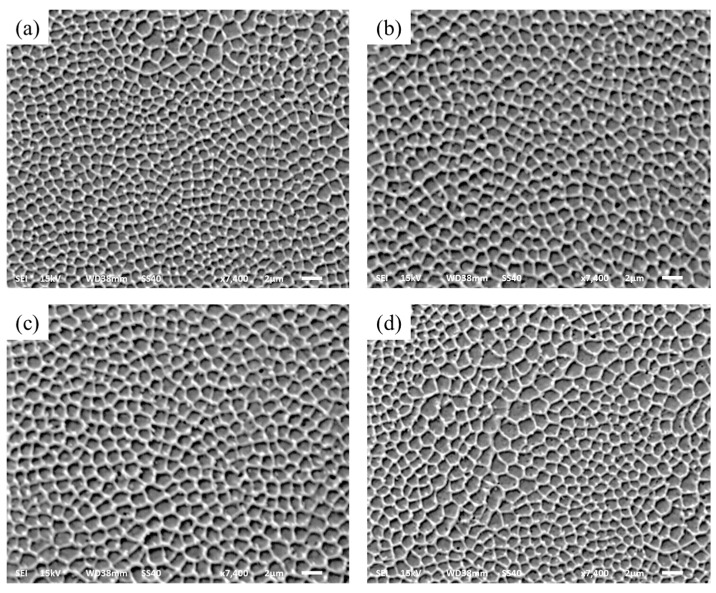
Microstructure of different exposure times (**a**) 80 μs; (**b**) 120 μs; (**c**) 160 μs; (**d**) 200 μs.

**Figure 11:**
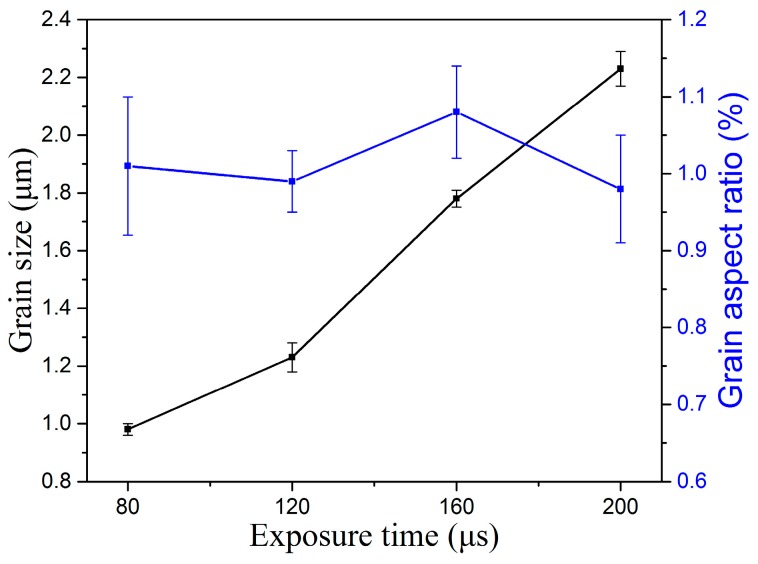
Grain size and aspect ratio of different exposure times.

During the SLM fabrication process, the microstructure is the main influence on exposure time, and can be a function of exposure time. The different exposure times result in the different microstructure. The cross section of the microstructure was observed when selecting the exposure times of 80, 120, 160, and 200 μs. The microstructures of different exposure times can be depicted as in Figure 10, and the graphical representation of these microstructure characteristics of the grain size and grain aspect ratio is shown in Figure 11. As the exposure times increase, the grain size grows slowly from 0.98 μm to 2.23 μm, and the grain size is very fine. Due to the spot-to-spot fabrication with a high degree supercooling of each molten pool, the microstructure is indicated by a mostly equiaxed crystal and a small amount of columnar crystal. Figure 11 also reflects the grain aspect ratio at the different exposure time; the grain aspect ratio indicates the difference in grain size along the horizontal and vertical directions. At exposure times from 80 μs to 200 μs, the grain aspect ratio is close to 1 and is indicated that the grain process isotropic mechanical properties in horizontal and vertical directions [42].

#### 3.2.5. Tensile Properties

**Figure 12:**
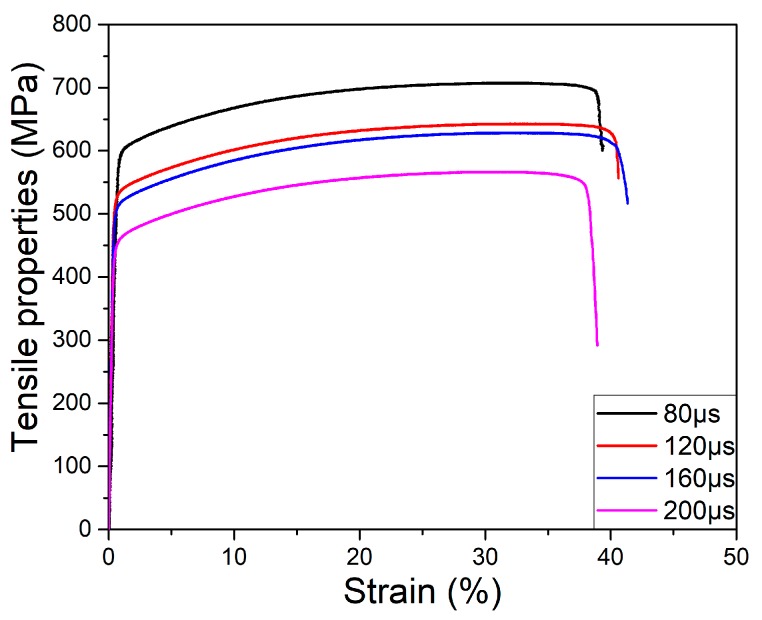
Tensile properties of samples with different exposure times of 80 μs, 120 μs, 160 μs, and 200 μs.

**Figure 13:**
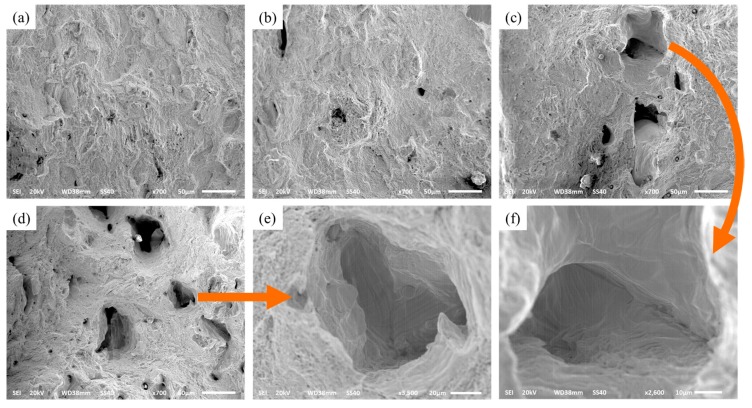
Morphology of tensile fracture surface (**a**) exposure time of 80 μs; (**b**) exposure time of 120 μs; (**c**) exposure time of 160 μs; (**d**) exposure time of 200 μs; (**e**) local enlarged image of coarse dimple rupture of (d); (**f**) local enlarged image of coarse dimple rupture of (c).

The tensile properties of different exposure times of 80, 120, 160, and 200 μs are shown in Figure 12, which possessed stable values of ultimate tensile strength (UTS) ranges from 550 MPa to 700 MPa, yield strength (YS) ranges from 450 MPa to 600 MPa, and elongation (EL) ranges from 39.7% to 41.8%. The average values of UTS, YS, and EL are 625 MPa, 525 MPa, and 39.9%, respectively. The exposure time of 80 μs possesses the highest UTS, YS, and a maximum EL of 160 μs. The SEM-fracture morphologies of tensile properties can be depicted as in Figure 13. Obviously, all of the fracture presents a dimple rupture, and the dimple becomes coarse as the exposure times increase. The exposure time at 80 μs contains a finer dimple rupture, comparing the 200 μs with the coarser dimple rupture. Figure 13e,f is the morphology of dimple rupture with 200 μs and 160 μs, respectively. Coarse dimple rupture leads to poor tensile properties easily, and a fine dimple rupture possesses little damage to the tensile properties. In addition, tensile properties are consistent with the microstructure. A finer equiaxed crystal can be obtained at exposure time 80 μs, resulting in a maximum of UTS and YS, and the tensile properties decrease as the grain size increases. The mechanical properties of metal are directly affected by average grain size from the Hall-Petch equation [43,44]. Also, the fine grain can improve the tensile properties, and the coarse grain results in poor tensile properties easily. Grain sizes depend on the fabrication process parameters; reducing the exposure time can dominate the molten pool with a high nucleation rate and supercooling.

Table 6 shows the tensile properties of different layer thicknesses; it indicates that the high layer thickness of 150 μm also can obtain excellent mechanical properties. There is no significant difference in the mechanical properties compared with the low layer thickness fabrication. It is due to the same metallurgical bonding and microstructure.

## 4. Conclusions

Selective Laser Melting of 316L has been carried out in this research. The experimental approach with the single-track experiment and multi-layer fabrication was involved, and the influences of process parameters on relative density, defect, microstructure, and tensile properties were analyzed at a high layer thickness of 150 μm and a fine powder of average particle (D_50_) of 18 μm. The obtained conclusions are as follows:

1. Single-track experiments with different combinations of exposure times and point distances can effectively narrow down the selection windows.
2. Multi-layer fabrications can be obtain approximation full-relative-density block ranges from 99.53% to 99.99%. The best relative density value can be reached at 99.99% when the process parameter of exposure time is 120 μs, the point distance is 40 μm, and the hatch space is 240 μm. The building rate can be up to 12 mm^3^/s, which is about 3 to 10 times higher than the previous studies.
3. There are three typical defects: the un-melted defect between the molten pools, the micro-pore defect within the molten pool, and the irregular distribution of the splashing phenomenon. The un-melted defect, that has a huge influence on the relative density, can be completely eliminated by adjusting the process parameters. Micropore and splashing were caused by hollow powder, circulating gas, and splash slag with smaller influence on the relative density, which can be partly reduced (it is difficult to completely eliminate it only through adjusting process parameters).
4. The microstructure of SLM high layer thickness fabricated indicates mostly fine equiaxed crystals and a small amount of columnar crystals because of a single molten pool with high degree of the subcooling. The average values of UTS, YS, and EL are 625 MPa, 525 MPa, and 39.9%, respectively, which are not significant difference in previous studies.

## Figures and Tables

**Table 1:** Chemical composition of 316L.

Element	Fe	Cr	Ni	Mo	Mn	Si	N	O	P	C	S
wt %	Balance	16–18	10–14	2–3	2	1	0.1	0.1	0.045	0.03	0.03

**Table 2:** Single-track experiments of process parameters.

Parameter	Value	Increment
Laser Power	380 W	-
Exposure time	80–200 μs	40 μs
Point distance	20–50 μm	10 μm
Layer thickness	150 μm	-
Laser beam spot size	150 μm	-
Atmosphere	Oxygen target <200 ppm	-

**Table 3:** Multi-layer fabrications of process parameters.

Exposure Time (μs)	Point Distance (μm)	Hatch Space (μm)			
80	20	120	160	200	240
120	40	160	200	240	280
160	30	200	240	280	320
200	50	240	280	320	360

**Table 4:** Summary of different particle sizes in previous studies.

Layer Thickness (μm)	Particle Size of D_50_ (μm)	Relative Density (%)	References
30	42	99.00	Kamath [20]
50	27	98.60	Zhang [35]
50	30	99.62	Cherry [21]
50	42	99.88	Sun [22]
80	36	99.80	Ma [23]
150	18	99.99	In this research

**Table 5:** Summary of the building rate with previous studies.

Layer Thickness [μm]	Hatch Space [μm]	Scanning Speed [mm/s]	Exposure Time [μs]	Building Rate [mm^3^/s]	References
30	55	1600	-	2.64	Kamath [20]
50	124	-	125	2.48	Cherry [21]
30	450	15	-	0.90	Zietala [38]
50	110	-	80	4.13	Casati [17]
50	35	1500	-	1.67	Sun [22]
100	3000	8.34	-	3.60	Guo [39]
150	240	-	120	12.00	In this research

**Table 6:** Summary of Tensile properties with different layer thicknesses.

Layer Thickness (μm)	UTS (MPa)	YS (MPa)	EL (%)	References
50	625	547	18	Zhang [35]
60	653	534	16	Mertens [45]
100	770	415	40	Guo [39]
50	684	554	36	Casati [17]
30	680	520	25	Suryawanshi [36]
150	625	525	39.9	In this research
